# When to Trust Epigenetic Clocks: Avoiding False Positives in Aging Interventions

**DOI:** 10.1111/acel.70756

**Published:** 2026-10-08

**Authors:** Daniel S. Borrus, Raghav Sehgal, Jenel Fraij Armstrong, Jessica Kasamoto, John Gonzalez, Albert Higgins‐Chen

**Affiliations:** ^1^ Department of Psychiatry Yale University School of Medicine New Haven Connecticut USA; ^2^ Program in Computational Biology and Bioinformatics Yale University New Haven Connecticut USA; ^3^ Department of Pathology Yale University School of Medicine New Haven Connecticut USA

**Keywords:** anti‐aging, biomarkers, epigenetic clocks, interventions, longevity

## Abstract

Epigenetic clocks have emerged as potential biomarkers of aging due to their association with morbidity and mortality. They have recently gained traction as outcome measurements for anti‐aging interventions. However, as the number of new epigenetic clocks continues to grow, different clocks may not agree on the effect of an intervention. This raises the question of when researchers can trust epigenetic clock results. In this study, we find that when extending previously published single‐clock intervention studies to include additional clock models, no additional clocks reach statistical significance. We then analyze clock behavior across positive control datasets (age‐accelerating events) to propose three criteria that increase confidence in a result: (1) reliable PC‐based clocks respond, (2) mortality‐trained or pace‐of‐aging clocks respond, and (3) multiple clocks respond in the same direction. To explain why genuine effects would appear across multiple clocks, we perform a simulation study to demonstrate how reliable clocks are more sensitive to true effects, and use longitudinal control datasets to show that reliable clock changes are correlated with each other. We then show examples of interventions that do satisfy these criteria. These findings provide a framework for interpreting epigenetic clock results in anti‐aging intervention studies, and suggest that some previously published single‐clock findings may warrant reexamination.

## Graphical abstract

When only one epigenetic clock responds to an intervention, noise and clock‐specific biology predict the same result. We propose three criteria that increase confidence in a reported change: reliable PC‐based clocks respond, mortality‐trained or pace‐of‐aging clocks respond, and multiple clocks respond in the same direction.

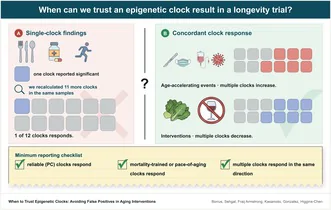

## 1 Introduction

In the pursuit of extending human healthspan, various interventions are being developed to target the underlying biological mechanisms associated with aging, including dietary regimens, supplements, and pharmaceutical agents (López‐Otín et al. 2023; Rolland et al. 2023). The primary aim of these interventions is to reduce age‐related morbidity or mortality and to maintain function. Ideally, such interventions begin long before pathology leads to a notable decline. However, clinical trials spanning the many years or decades needed to observe the effect on human aging would be very difficult and expensive. Aging biomarkers have been proposed as a means for researchers to assess the impact of specific interventions within a feasible time frame for clinical studies (Moqri et al. 2023; Aging Biomarker Consortium et al. 2023). Such biomarkers are trained to quantify biological age or pace of aging as a proxy for longer‐term outcomes. However, research into how these biomarkers respond to interventions, and the significance of observed biomarker changes, remains in its infancy.

Epigenetic clocks are aging biomarkers based on DNA methylation at cytosine‐guanine dinucleotides (CpGs). These clocks have gained significant popularity over the past decade due to their prognostic power and the ease and speed of measurement, requiring a simple blood draw (Horvath and Raj 2018; Drew 2022). The first generation of epigenetic clocks, such as the Hannum, Horvath multi‐tissue (Horvath MT), and Horvath skin‐and‐blood (Horvath SB) clocks, utilized penalized regression models (e.g., elastic net) to predict chronological age from DNA methylation patterns (Hannum et al. 2013; Horvath 2013; Horvath et al. 2018). Newer generations of clocks use similar techniques but are trained to predict mortality and morbidity risk; these include PhenoAge and GrimAge (Levine et al. 2018; Lu et al. 2019). Another recent clock model, DunedinPoAm38, was trained on longitudinal biomarkers to predict an individual's pace of aging (Belsky et al. 2020). These models also differ in the tissue they were trained on: Hannum, PhenoAge, GrimAge, and the Dunedin measures were all trained in whole blood, whereas the Horvath MT clock was trained across more than fifty tissue and cell types and the Horvath SB clock on skin, blood, and cultured cells. Many of these epigenetic‐based measurements of biological age have been shown to be prognostic, correlating at least partially with outcomes such as mortality (Simpson and Chandra 2021).

Beyond predictive capabilities, epigenetic clocks may be used in a clinical trial setting to rapidly calculate an individual's biological age before and after an aging intervention and assess the impact of treatment. Epigenetic clocks have already been applied in this fashion for several interventions, such as diet, exercise, and supplements (Sae‐Lee et al. 2018; Gensous et al. 2020; Fitzgerald et al. 2021; Yumi Noronha et al. 2022). However, there are several potential issues with using epigenetic clocks as metrics in longitudinal trials.

The first potential problem with measuring the impact of an intervention on epigenetic age is longitudinal reliability. We previously demonstrated that re‐testing the same individual can lead to fluctuations between 0.9 and 2.4 years, owing to technical noise and other confounders (Higgins‐Chen et al. 2022). This concern led us to develop PC clocks, re‐trained versions of the canonical clocks that use principal component analysis to reduce the effect of noise from individual CpGs, decreasing the median deviation of technical replicates to 0.3–0.8 years (Higgins‐Chen et al. 2022). Similarly, DunedinPACE is a modified version of the DunedinPoAm38 pace‐of‐aging predictor that increases reliability and longitudinal performance by only utilizing reliable CpGs as input (Belsky et al. 2022). However, while the improved reliability may reduce false positives, it does not fully address the question of which clocks to use or how to interpret disagreement between clock models.

This leads to a second practical issue: with an abundance of clock models, which one should a researcher select for their particular study? How can we be sure which clock is the most relevant? And if multiple clocks are calculated, how do we interpret the situation where different clocks disagree? This is an ongoing problem in the literature—several studies that use epigenetic clocks to measure an aging intervention report results from a single clock model in their analysis but it is unclear if the chosen clock is most appropriate (Sae‐Lee et al. 2018; Gensous et al. 2020; Fitzgerald et al. 2021; Yumi Noronha et al. 2022). Under these conditions, the field is at risk for publication bias, opting for clocks that return significant results and ignoring the non‐significant results. A direct comparison of the responsiveness of the various clocks to aging interventions is warranted.

An epigenetic clock measurement contains three components: a shared signal reflecting systemic biological aging, a clock‐specific component reflecting each model's training tissue and outcome, and some technical noise. The overlapping/shared component causes the clocks to correlate with one another (they are all, to some degree, related to aging). The clock‐specific component leads to some amount of differential response or specificity to an anti‐aging intervention. Technical noise obscures the true signal and is the problem the reliable clocks were built to address.

We hypothesize that sporadic single‐clock findings are more likely to reflect noise or a clock‐specific effect than a systemic change in biological aging, which should be detectable across multiple clock models. To investigate this, we analyze 12 epigenetic clocks (6 well‐established clocks and their high‐reliability PC counterparts) across multiple longitudinal DNA methylation datasets. We first re‐examine previously published intervention studies that reported single‐clock significance, testing whether additional clocks corroborate the original findings—specifically, did any additional clock also show a statistically significant change in the same direction? We then turn to positive control datasets capturing age‐accelerating events (intensive surgery, cancer treatment, COVID‐19 infection), reasoning that if clocks can detect decreases in biological age, they should also detect increases. From these positive controls, we derive three criteria for increased confidence in an epigenetic clock result. We perform simulation studies to demonstrate why reliable clocks are more sensitive to true effects, and longitudinal control datasets to show that clock changes are correlated across models. We apply these insights to candidate anti‐aging interventions and identify three datasets that do meet these criteria. Finally, we show that a clock average or, in the most stringent case, multiple hypothesis testing can be used as a simple filter for clock response. Together, these analyses provide a framework for interpreting epigenetic clock changes in intervention studies.

## 2 Results

### 2.1 Extending Intervention Analysis With Additional Clock Models Reveals Contradictory Results

We identified three intervention datasets (GSE149747, E‐MTAB‐8956, and GSE74548) that showed a significant decrease in biological age as measured by an epigenetic clock after an aging‐targeted intervention. Two of these studies, GSE149747 (an 8 week diet, exercise, and supplement intervention) and GSE74548 (a 2 year folic acid and vitamin B12 supplement intervention), published previously that their interventions significantly decreased biological age as measured by the original Horvath MT clock. More details on the specific datasets can be found in Table 1.

**TABLE 1:** Twelve longitudinal human DNA methylation datasets that capture an aging intervention, aging event, or act as control datasets.

Accession #	Intervention/Event type	Duration	*N* subjects	Baseline age	Tissue type	Array	Array missingness (%)	Normalization method
GSE149747 (Fitzgerald et al. 2021)	Diet, Supplements, Exercise, and Lifestyle	8 weeks	19	57.8 ± 6.3 (50–72)	Saliva	EPICv1	0.339	Illumina default only
E‐MTAB‐8956 (Yaskolka Meir et al. 2016; Gepner et al. 2018)	Low carb diet and exercise	1.5 years	30	49.7 ± 9.8 (30–65)	Whole blood	EPICv1	5.037	Noob + quantile
GSE74548 (Sae‐Lee et al. 2018)	Folic acid and vitamin B12 supplements	2 years	14	70.7 ± 3.3 (66–75)	Buffy coat	450 K	0.637	SWAN
GSE142536 (Sadahiro et al. 2020)	Intensive surgery	1 day	30	77.9 ± 6.4 (62–91)	PMBC	450 K	0.919	Dasen (background adjustment + between‐array quantile)
GSE140038 (Sehl et al. 2020)	Radiotherapy with or without chemotherapy	Months	72	56.7 ± 10.2 (36–77)	Whole blood	EPICv1	0.339	Noob
GSE219037 (Mao et al. 2023)	SARS‐CoV‐2 virus infection	1–2 months	35	19.7 ± 2.4 (18–27)	Whole blood	EPICv1	18.798	ChAMP + ComBat
GSE123914 (Zaimi et al. 2018)	Control	1 year	34	58.4 ± 3.5 (51–64)	Whole blood	EPICv1	8.567	Funnorm + ComBat
GSE61151 (Flanagan et al. 2015)	Control	5–7 years	92	53.2 ± 8.6 (35–77)	Whole blood	450 K	0.766	Illumina default only
GSE73115 (Tan et al. 2016)	Control	10 years	86	76.2 (73–82)	Whole blood	450 K	0.637	Illumina default only
GSE98876 (Brückmann et al. 2017)	Alcohol Treatment Program	3 weeks	24	47.5 ± 10.1 (26–67)	CD3+ T‐cells	450 K	0.637	Illumina default only
E‐MTAB‐12527 (Yaskolka Meir et al. 2021)	Mediterranean diet with red meat	2 years	81	51.8 ± 10.5 (34–76)	Whole blood	EPICv1	6.233	Noob + quantile
E‐MTAB‐12527 (Yaskolka Meir et al. 2021)	Mediterranean diet with no red meat	2 years	87	50.8 ± 11.0 (32–82)	Whole blood	EPICv1	6.233	Noob + quantile

To broaden the measurement of the intervention response, we extended those analyses by calculating 11 additional epigenetic clock models, including generation 2 mortality‐based epigenetic clocks (PhenoAge and GrimAge) as well as pace‐of‐aging clocks such as DunedinPoAm38 and DunedinPACE. We performed a Student's paired *t*‐test on epigenetic age residual (Methods) before and after the intervention for each of the 12 epigenetic clocks (Figure 1). In datasets which had control cohorts, we also calculated unpaired *t*‐tests between the subject and control groups' age residual (Figure S1).

**FIGURE 1:**
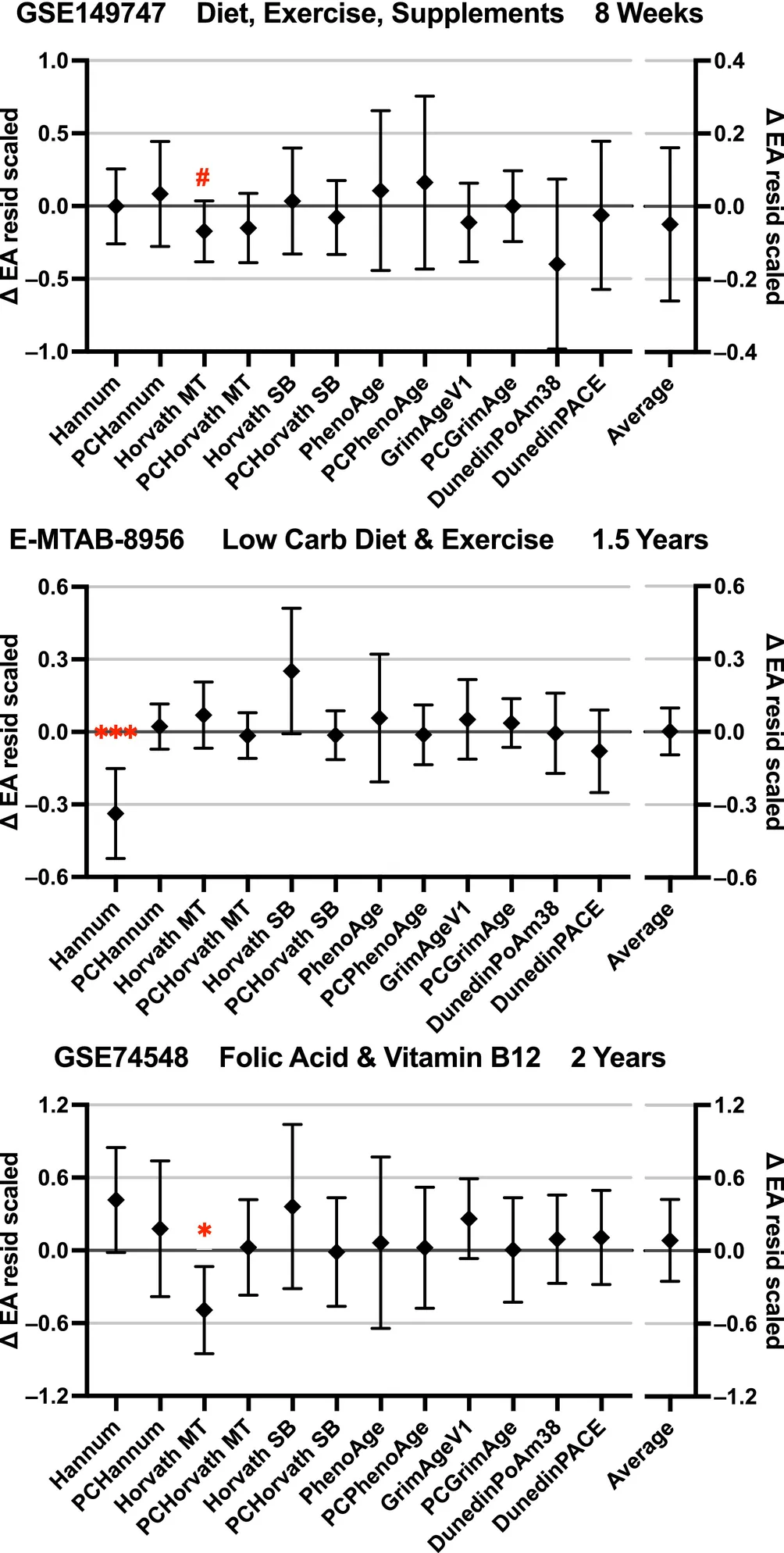
Epigenetic clock models measuring three different aging interventions. Black diamonds show the mean change in epigenetic age residual (∆ EA resid scaled). Values are scaled to the clock standard deviation in a reference dataset. The average change across all 12 clocks is shown on the right of each graph. Error bars indicate 95% confidence intervals. * indicates *p* < 0.05. *** indicates *p* < 0.001 Red hash for GSE149747 indicates a *p*‐value of 0.09.

Among the 11 new epigenetic clock measurements, none changed significantly in the same direction as the originally reported result. We found no significant response even when comparing the more reliable PC versions to the original clock (i.e., PCHorvath MT vs. Horvath MT) (Figure 1). This lack of agreement across clocks raises questions about the reliability of these single‐clock findings: are the single clock results measuring unique aspects of biological aging or are they statistical artifacts?

### 2.2 Positive Control: Age‐Accelerating Events Are Captured by Multiple Reliable Clocks

To better understand what pattern of clock changes would indicate a genuine shift in biological age, we turned to positive control datasets where aging effects are expected. We reasoned that if it is possible to measure an intervention that decreases biological age, then the reverse should also be true: events known to increase mortality should result in measurable biological age acceleration. Indeed, a prior study showed that stressful events (surgery, pregnancy, severe COVID‐19) lead to strong but reversible increases in epigenetic age according to multiple clocks (Poganik et al. 2023). We reasoned that we could treat these age‐accelerating events as positive controls. By observing their effects on multiple epigenetic clocks, we can gain insight into what would constitute a trustworthy pattern of epigenetic clock change in response to aging interventions. With this hypothesis in mind, we repeated our 12‐clock analysis on three longitudinal datasets that captured events with a known association with mortality (Figure 2, Table 1). We examined epigenetic clocks before and after intensive surgery (GSE142536, previously analyzed by Poganik et al. (Poganik et al. 2023)), before and after radiation and chemotherapy (GSE140038, not previously analyzed), and before and after SARS‐CoV‐2 infection (GSE219037, not previously analyzed).

**FIGURE 2:**
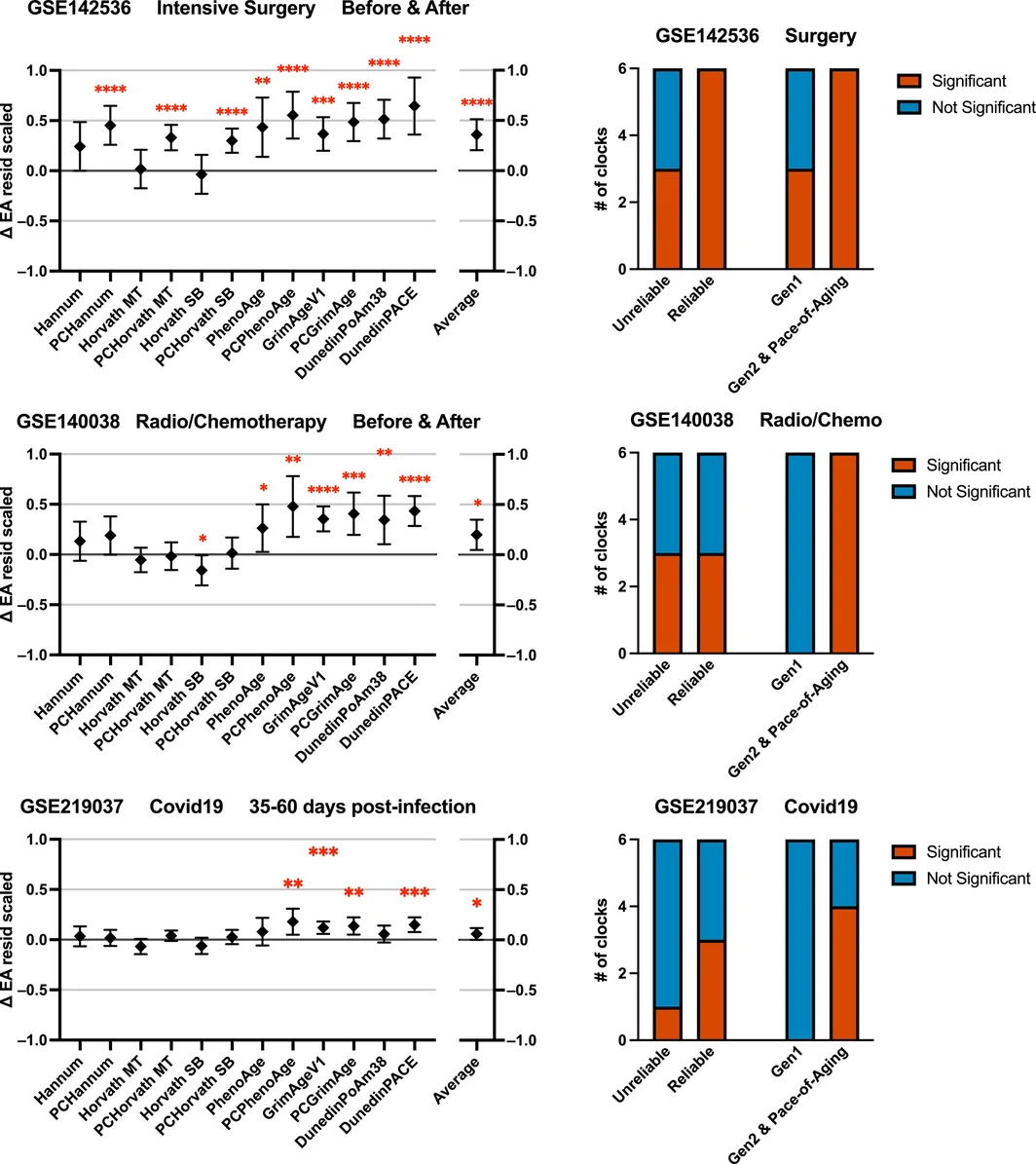
Three positive control methylation datasets. Each dataset captures an age‐accelerating event. Average effect sizes, as measured by 12 epigenetic clocks, are scaled with a reference dataset and are shown. Error bars indicate 95% confidence intervals. Red asterisks show the significance of the change: *p < 0.05, **p < 0.01, ***p < 0.001, ****p < 0.0001. On the right, a count of significantly increased clocks, categorized by reliability or generation.

In all three datasets, we found significant increases in at least 4 of the 12 clocks tested (Figure 2). Notably, PCPhenoAge, PCGrimAge, and DunedinPACE (three clocks which are highly associated with mortality) significantly increased in all three event datasets. To summarize this agreement while filtering out clock‐specific differences and technical noise, we computed a per‐subject 12‐clock average. This measurement is not a new biomarker, but a filter that quickly determines if the clocks generally agree. In each dataset, this 12‐clock average was significantly positive, indicating some level of concordant epigenetic age acceleration across clocks. This conclusion was insensitive to which clocks were included in the average; it held using the reliable clocks alone and in 137 of 144 leave‐one‐out tests (Figure S2; Table S1).

We observed clear trends by clock category. Reliable clocks were more likely to detect the age‐accelerating events than their non‐reliable counterparts. Pooling across datasets, 12 of 18 reliable clock tests reached significance compared to 7 of 18 for non‐reliable clocks. Similarly, Gen 2 mortality‐trained and pace‐of‐aging clocks outperformed Gen 1 chronological clocks (16 of 18 significant vs. 3 of 18).

The non‐reliable versions of the Gen 1 chronological‐based clocks (Hannum, Horvath MT, and Horvath SB) fail to report a significant increase in biological age in any of the three datasets. At worst, Horvath SB indicates a significant decrease in biological age after radiotherapy and chemotherapy.

Taken together, the positive control datasets offer three criteria for increased confidence in the interpretation of a change in biological age as measured by epigenetic clocks. In the ideal scenario, we can be more confident there was a measurable change in biological age when (1) the reliable PC‐based clocks respond, consistent with their reduced technical noise, (2) the Gen 2 mortality and Pace‐of‐Aging clocks respond, consistent with mortality risk and pace of aging changing with health status while chronological age does not, and (3) multiple clocks respond in the same direction, consistent with the shared component they hold in common.

### 2.3 Simulation Study: Decreased Noise Leads to Higher Sensitivity for Effect Size in Reliable Clocks

Reliable clocks were designed to reduce technical noise, resulting in higher intraclass correlation coefficients (ICCs, a measure of test–retest reliability). Higher biomarker ICC helps, in turn, to decrease error when measuring an individual's biological age longitudinally. The difference between a reliable and non‐reliable clock measuring a null or real effect size is visualized in Figure 3A. When there is no true effect size, that is, no change in biological age, the wider distribution of the non‐reliable clock has no impact on significance. However, when there is a true effect size, that is, a real change in biological age, the wider distribution leads to a lack of sensitivity for the true change. The reliable clock, with a narrower distribution of effect size, is more sensitive to the true change.

**FIGURE 3:**
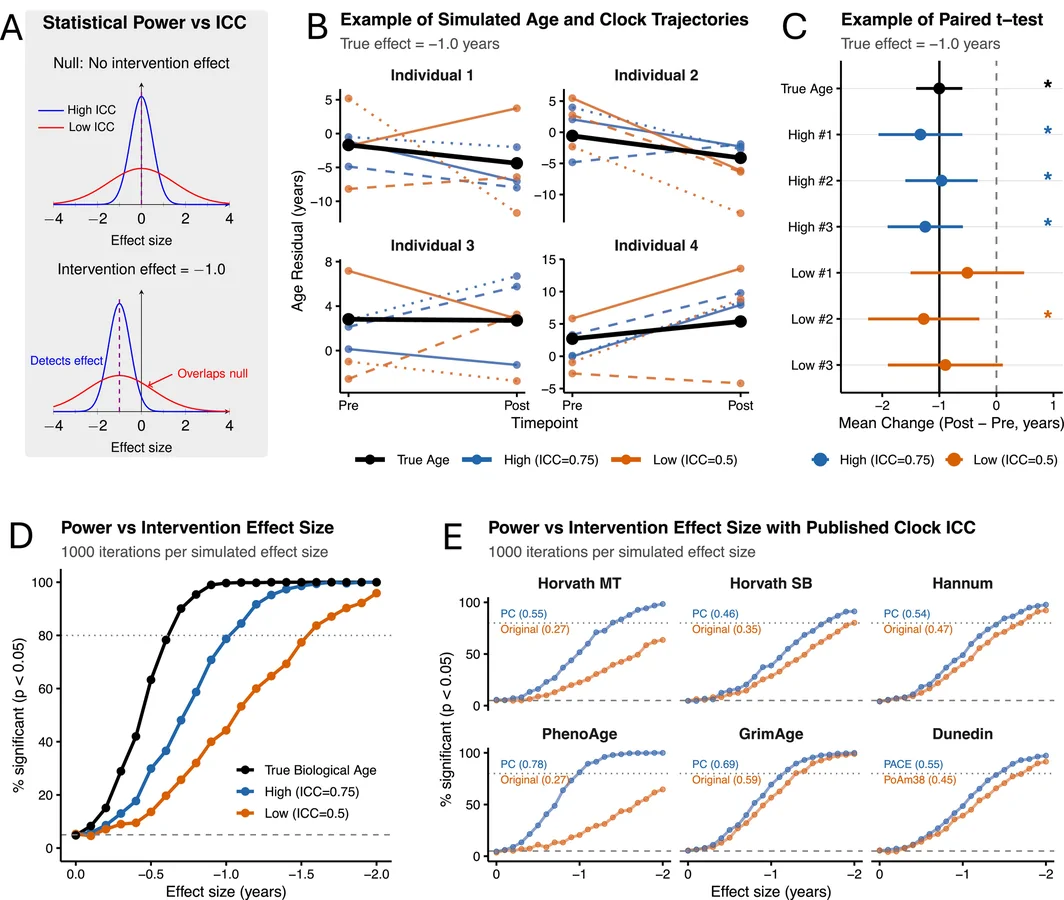
Simulation‐based power analysis of biomarker reliability and sensitivity to effect size. (A) Diagram depicting the impact of reliability on statistical power. (B) Example simulation of biological age and biomarker predicted ages with four individuals. (C) Example *t*‐test for a single simulated intervention. Error bars indicate 95% confidence intervals. (D) Parameter study of effect size on the percentage of significant clocks. Each point captures the average percentage of significant clocks across 1000 iterations. (E) Identical power analysis as in D, with clock ICCs determined by published ICC scores.

We verified this hypothesis with a simulation study of a hypothetical clinical trial. We generated a ground‐truth biological age trajectory for 200 individuals, across two timepoints, with an average decrease in biological age of −1 (Figure 3B; see Table 3 for full parameter set). For each individual, 6 epigenetic clock scores were derived: 3 with low ICC (0.5) and 3 with high ICC (0.75) (Figure 3B). Note that ICC here refers to test–retest reliability across multiple measurements across different time points, which is lower than the ICC for technical replicates. The variable ICCs represent clocks with different amounts of technical accuracy. During one simulated clinical trial, we calculated the change in clock score (and true biological age) between the two time points, and flagged significant decreases in predicted biological age across the population (Figure 3C). For incremental values of *δ* (effect size) ranging from 0 to −2, we repeated this clinical trial simulation 1000 times, and calculated the percentage of significant results for low and high ICC clocks. Figure 3D shows that as the magnitude of the effect size increases, the high reliability clocks detect the true change at a faster rate than the low reliability clocks. To validate these findings, we repeated this analysis with published clock ICCs (Figure 3E). Higgins‐Chen et al. (2022) calculated ICCs for the original and PC variants of Horvath MT, Horvath SB, Hannum, PhenoAge, and GrimAge from the Swedish Adoption/Twin Study of Aging (SATSA). Borrus et al. (2025) calculated ICCs for DunedinPACE and DunedinPoAm from a meta‐analysis of four biological reliability datasets. Due to higher ICC, reliable clocks detect true changes at more modest effect sizes, which is likely why they responded in the positive controls examples from the previous section.

### 2.4 Correlated Clock Trajectories Lead to a Multi‐Clock Coordinated Response

We hypothesized that multiple clocks should respond together to a genuine change in biological age, given the known correlation among clock residuals in cross‐sectional data. We sought to replicate this result and extend it to three longitudinal control datasets to ask: are the clock's longitudinal changes also correlated? We used control datasets, rather than the intervention (GSE149747, E‐MTAB‐8956, GSE74548) or the positive control datasets (GSE142536, GSE140038, GSE219037), to avoid confounding the trajectories by an experimental effect.

We calculated the cross‐clock correlation between the 12 epigenetic clock scores in three longitudinal control datasets (GSE61151, GSE73115, and GSE123914). Information such as follow‐up time and cohort size can be found in Table 1. We first calculate cross‐clock correlation on the age residuals of each dataset at the initial baseline timepoint, and then average the correlations from the three datasets together (Figure 4A; correlation results for individual dataset results are reported in Figure S3). As has been reported previously, there was a strong correlation in clock residuals, as each clock captures some overlapping aspect of biological aging (i.e., individuals that appear older using the Horvath clock tend to appear older in the PhenoAge clock).

**FIGURE 4:**
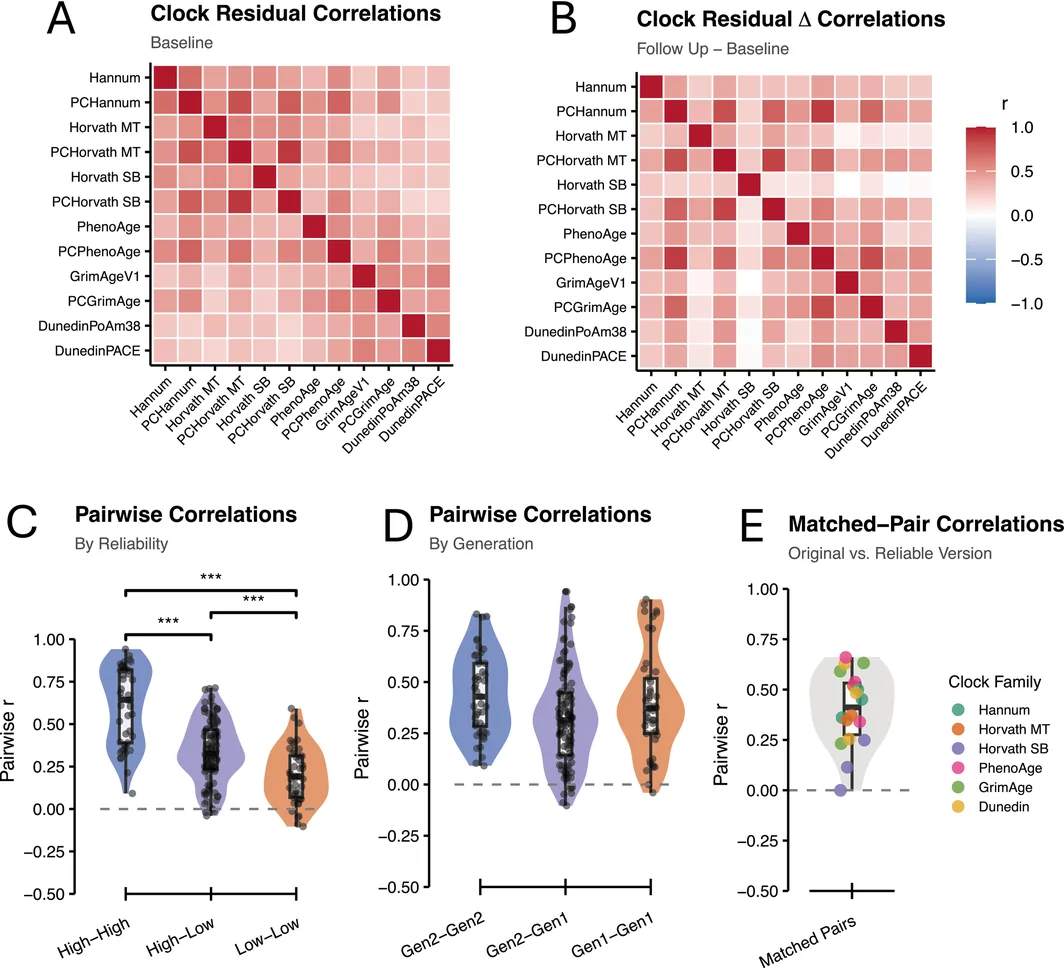
Cross‐sectional and longitudinal trajectory correlations between 12 epigenetic clocks. (A) Correlation (*r*) between 12 epigenetic clocks at baseline sample. *r* values shown here are averages from three control datasets. (B) Correlation (*r*) between longitudinal changes in clock scores from 12 epigenetic clocks. *r* values shown are averages from three control datasets. (C) Scatter plot showing pairwise correlations between clocks grouped by reliability. (D) Scatter plot showing pairwise correlations between clocks grouped by generation. (E) Matched‐pair correlations between original and reliable versions of each clock.

Next, we calculated the change in biological age residual from baseline sample to the follow‐up sample (residual Δ; Figure 4B). For each dataset, we calculated the cross‐clock correlation between the residual Δs and took the average across the three datasets (Figure 4B; correlations for individual dataset results are reported in Figure S3). Most clock trajectories were, in general, correlated with one another, as most of the individual correlations are positive (red). One outlier here is the original Horvath SB clock, which is visually the least correlated with the other clocks.

To measure the strength of correlations categorically, we compared the pairwise correlation between high reliability clocks (high‐high), low reliability clocks (low‐low), and between different groups (high‐low) (Figure 4C). The reliable clocks tend to change together, as seen by the increased pairwise correlation (high‐high mean *r* = 0.61 vs. low‐low mean *r* = 0.20, *p* < 0.001), suggesting that the inherent noise of the low reliability clocks is what leads them to change in spurious directions when the rest of the clocks tend to agree.

To rule out the possibility that the correlation between reliable PC clocks was driven by their underlying shared set of CpGs, we also looked at pairwise correlations between each PC clock and DunedinPACE and compared these to the pairwise correlations between DunedinPACE and the original clocks (Figure S4). We found DunedinPACE was less correlated with the original nonreliable clocks than the PC variant, indicating that the correlation between reliable clocks is not entirely driven by shared underlying CpGs.

We also categorized the pairwise correlation by clock generation (Gen 2—Gen 2, Gen 1—Gen 1, and Gen 2—Gen 1; Figure 4D) but found no significant difference, suggesting that clocks across generations tend to agree on changes in biological age in the absence of a targeted intervention.

We looked at the pairwise correlations between the reliable and non‐reliable versions of a single clock (Figure 4E) to see if any particular clock was discordant with its reliable counterpart. Five of the matched pairs showed moderate positive correlations in the aggregate (mean *r* = 0.41–0.51), with the exception of Horvath SB, which showed weak correlation with its PC counterpart (mean *r* = 0.12).

The correlation between clock Δs (Figure 4B) suggests that an average of the clock changes is a valid way to measure consensus about biological age change. Because the clocks tend to change together, averaging across clocks reduces measurement error at the risk of dampening signals with less responsive clocks. To validate this approach, we calculated the average change across all 12 clocks for each individual in each dataset analyzed thus far. The single‐clock responder datasets (Figure 1) do not show a significant average clock change, whereas all three positive control datasets (Figure 2) do show a significant positive change. This reaffirms the hypothesis that the clock average can serve as a simple, interpretable filter for detecting systematic shifts in epigenetic age.

### 2.5 True Positives: Validated Lifestyle Interventions Modify Reliable and Gen 2 Clocks

We have shown that events that change biological age should (1) present in multiple clocks, due to the clock's longitudinal correlation, (2) present in reliable clocks, due to their decreased longitudinal noise, and (3) present in Gen 2 and pace‐of‐aging clocks, likely due to those clocks' sensitivity for biological age versus chronological age. We ask, are there examples of anti‐aging intervention datasets that satisfy all three of these requirements?

We identified two datasets which do satisfy all three of these conditions. The first is a 2‐year diet trial (E‐MTAB‐12527) with two separate arms; one cohort ate a standard Mediterranean‐style diet (MED) while the other cohort ate a Mediterranean diet with more red meat restrictions and enriched with green plants and polyphenols (green) (Figure 5). The second intervention dataset was a three‐week long alcohol treatment program (GSE98876), where chronic alcohol users abstained from alcohol use during the trial.

**FIGURE 5:**
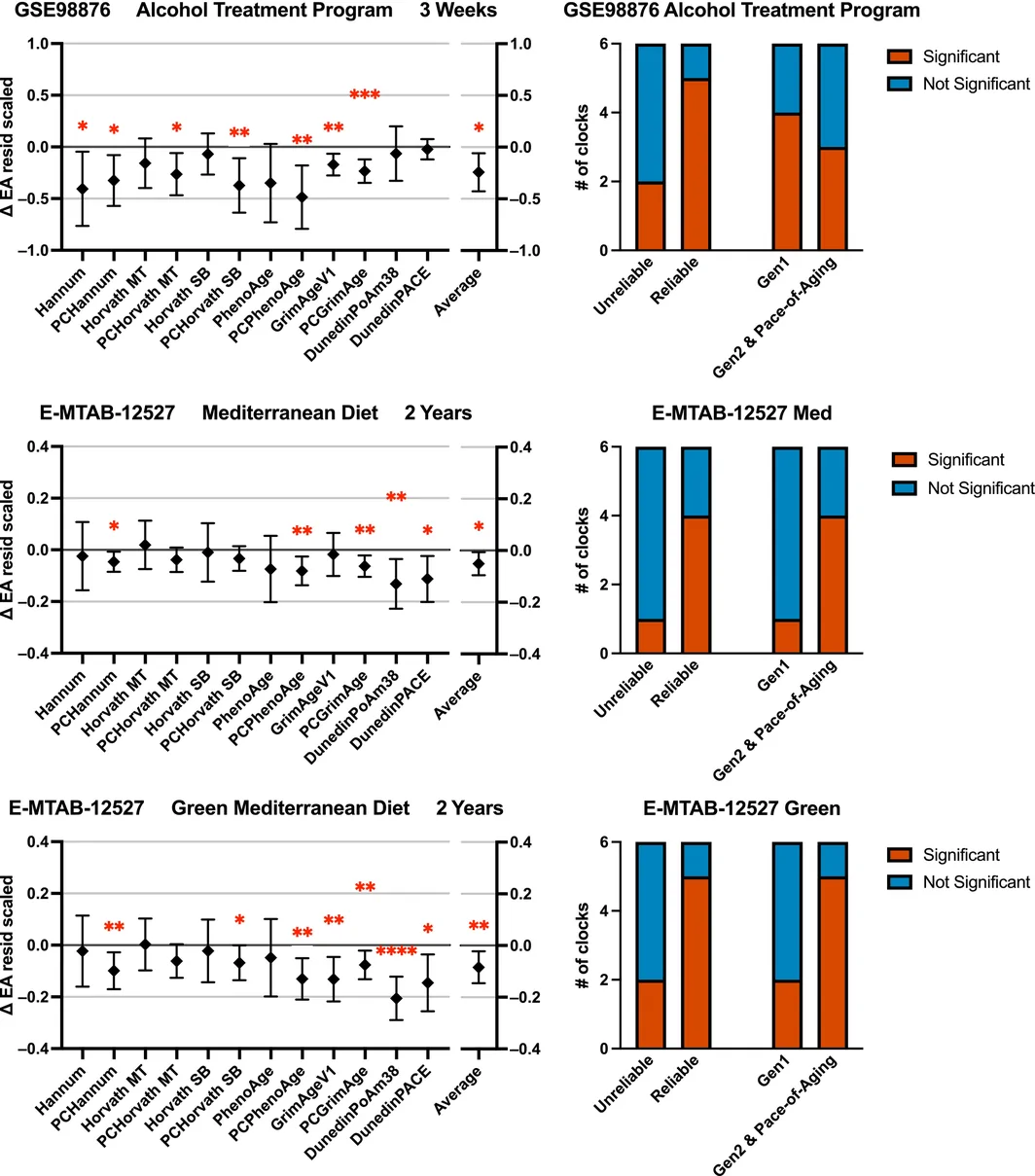
Three intervention datasets showing significant clock decreases. Average effect sizes, as measured by 12 epigenetic clocks, are scaled with a reference dataset and are shown. Error bars indicate 95% confidence intervals. On the right, a count of significantly decreased clocks, categorized by reliability or generation.

We repeated our 12‐clock analysis for all three interventions and compared clock scores before and after treatment. In each of the studies, at minimum five clocks responded with a significant decrease, with seven clocks responding in both the alcohol treatment program and the green Mediterranean diet.

In each of the three interventions, reliable clocks were more likely to respond with a significant change in biological age. Pooling the three datasets, just 5 of the 18 non‐reliable clocks significantly decreased, compared to 14 of the 18 reliable clocks. A similar story occurred with the move from Gen 1 clocks (7 out of 18 clocks decreased significantly) to Gen 2 and Pace‐of‐Aging clocks (12 out of 18 clocks decreased significantly). Interestingly, at nominal significance this trend was not observed in the alcohol treatment intervention (four Gen 1 vs. three Gen 2/Pace‐of‐Aging clocks); however, after multiple‐hypothesis correction only Gen 2 clocks (PCPhenoAge, GrimAge, PCGrimAge) remained significant (Table S2). The per‐subject average change across all 12 clocks is also significantly decreased in all three datasets, providing further evidence of a decrease in epigenetic age over the course of the intervention.

## 3 Discussion

Epigenetic clocks represent a promising biomarker candidate for measuring the impact of an anti‐aging intervention. However, with so many clocks to choose from, interpreting the results of a given study runs the risk of selection and publication bias. When can we trust the results of anti‐aging interventions measured with epigenetic clocks?

In this work, we propose three criteria for increased confidence in an intervention result: (1) reliable PC‐based clocks respond, (2) mortality‐trained or pace‐of‐aging clocks respond, and (3) multiple clocks respond in the same direction. We further show that an average across‐clock model provides a simple, interpretable metric for detecting shifts in epigenetic age. Datasets that meet these criteria are more likely to reflect bona fide changes in biological aging.

Our finding that Gen 1 chronological clocks (Hannum, Horvath MT, Horvath SB) are the last to agree with the rest of the clocks about a change in biological age is not surprising when considering how these clocks were trained. By optimizing prediction of chronological age, these models prioritize methylation sites that track time consistently and may be inherently desensitized to changes responsive to lifestyle, diet, or pharmacological interventions. This does not mean Gen 1 clocks are more prone to false positives; rather, they are less likely to detect genuine changes in biological aging because chronological age is not the outcome that interventions target.

In contrast, Gen 2 clocks trained on mortality (PhenoAge, GrimAge) and pace‐of‐aging clocks (DunedinPoAm38, DunedinPACE) are more responsive to intervention effects because they were trained on biologically meaningful outcomes. These clocks prioritize methylation sites associated with health and longevity, making them better suited for detecting the types of changes that anti‐aging interventions aim to produce.

Reliable PC‐based clocks demonstrate increased sensitivity to true effects due to their reduced longitudinal noise. As our simulation study shows, clocks with higher test–retest reliability detect true changes at more modest effect sizes. This explains why PC versions consistently outperform their non‐PC counterparts in our analyses. The non‐PC clocks are not more likely to produce false positives; rather, their intrinsic noise obscures the real signal.

The twelve clocks examined here are not interchangeable biomarkers. They were trained with different methodologies and on different definitions of biological age. Unsurprisingly, the field has shown moderate correlation between different clocks: GrimAge and PhenoAge age acceleration correlate at *r* = 0.45 (Lu et al. 2019), or *r* = 0.44 in our data (Figure 4), and DunedinPACE correlates with PhenoAge and GrimAge at *r* = 0.36 and *r* = 0.58 respectively (Belsky et al. 2022), or *r* = 0.38 and *r* = 0.62 in our data (Figure 4). Some discordance between clocks is therefore not automatically evidence of noise, as an intervention may impact one aspect of aging biology more than another. These correlations are partially attenuated by measurement error: the reliable PC versions of the same clocks agree substantially better (PCGrimAge‐PCPhenoAge *r* = 0.63 at baseline and *r* = 0.83 for longitudinal change vs. *r* = 0.44 and *r* = 0.41 for the originals), indicating that a portion of the apparent divergence is technical rather than biological. The challenge with a lone significant clock remains that clock‐specific biology and statistical noise predict the same observation, and the pattern alone cannot distinguish them. Attributing a solitary finding to that clock's specific biology therefore requires additional validation such as a mechanistic rationale or finding replication.

The risk of publication bias in epigenetic clock studies is not merely theoretical. In this work, we re‐analyzed datasets from previously published intervention studies that reported significant single‐clock findings, including GSE149747 and GSE74548 (Fitzgerald et al. 2021; Sae‐Lee et al. 2018). When we extended these analyses to include additional clock models, no additional clock corroborated the original findings. In GSE74548, ten of the eleven additional clocks trended in the opposite direction, and in GSE149747, the originally reported effect did not itself reach significance in our reanalysis. These results suggest that some published claims of epigenetic age reduction may warrant re‐examination.

This concern is amplified by the rapid growth of epigenetic clock usage in intervention research. As these biomarkers gain popularity as outcome metrics for anti‐aging treatments, the need for standardized reporting and interpretation becomes critical. Without clear criteria for what constitutes a trustworthy result, the field risks accumulating a literature of unreplicable findings.

Based on our analyses, we recommend that intervention studies report results from multiple epigenetic clock models and calculate a clock average per‐subject as a summary metric. Several studies have already adopted this multi‐clock approach: the impact of caloric restriction and umbilical cord plasma transfusions on biological age have both been investigated using multiple epigenetic clocks, providing more nuanced interpretations of intervention effects (Waziry et al. 2023; Clement et al. 2022). However, this practice is not yet the norm. Even recent studies that calculate multiple clocks often interpret a single positive result from a chronological clock while ignoring null results from mortality‐trained or pace‐of‐aging clocks (Gensous et al. 2020; da Silva Rodrigues et al. 2024; Patterson et al., n.d.). Our three criteria provide a framework for avoiding this selective interpretation.

We also examined whether multiple hypothesis correction could serve as an additional filter for distinguishing true from false positives (Table S2). Results were mixed: among the single‐clock responder datasets, one (GSE74548) lost significance after Bonferroni correction, while another (E‐MTAB‐8956) remained significant. The positive control and true positive datasets largely retained significance, particularly for high‐reliability mortality clocks such as PCGrimAge and DunedinPACE. However, given the correlation between clock changes demonstrated in Figure 4, strict multiple testing correction may be overly conservative; the clocks are not independent measurements, and thus Bonferroni in particular may overcorrect. For practical purposes, the three criteria and clock average described in Results may provide more consistent and interpretable guidance than formal multiple hypothesis correction alone.

An additional dimension for interpreting clock change is the contribution of cell‐composition changes. We re‐residualized each clock against age plus the 12 EpiDISH cell fractions and recomputed paired *t*‐tests on four intervention and three event datasets (Figure S5; Table S3). Alcohol treatment (GSE98876) and 8 week Diet, Exercise, Supplements intervention (GSE149747) were held out from the cell fraction deconvolution experiments as the samples were derived from purified CD3+ cells and saliva, respectively. The remaining effect size after adjusting for cell fractions should represent the epigenetic changes in biological age that are not related to cell composition changes. Attenuation of the median effect size varied across datasets. In the two datasets where effect sizes were already minimal (E‐MTAB‐8956 and GSE74548), we observed no systematic change in results. Surgery (GSE142536) and chemo‐radiotherapy (GSE140038) showed the greatest reduction in effect size (77% and 73% median attenuation across clocks, respectively). The SARS‐CoV‐2 dataset (GSE219037) was essentially unchanged after adjustment (0% median attenuation), and both arms of the Mediterranean‐diet study (E‐MTAB‐12527) retained most of their effect (31% and 34% median attenuation across clocks for the green‐Mediterranean and standard Mediterranean arms, respectively). This heterogeneity in signal attenuation indicates that some intervention effects are dominated by composition shifts, while others reflect cell‐independent CpG‐level change. This finding reaffirms why cell‐type decomposition is an important step in understanding epigenetic age changes in longitudinal datasets, but it does not invalidate the original results: clocks are trained on bulk blood and their validated predictive power depends on cell fraction changes. A full exploration of the impact of cell fraction changes on a broader set of clock performance metrics would be a valuable direction for future work.

Another concern is whether probe missingness could bias the comparison between clocks. Because the clocks draw on different CpG sets, the fraction of probes requiring imputation varies across clocks and datasets, from 0% for most clocks on 450 k arrays to as high as 28% for small clocks such as DunedinPoAm38 (Table S4). We explored the relationship between clock effect sizes and % of missing probes (within‐dataset *ρ* = −0.05; Figure S6) and found no clear trend, indicating that imputation was not driving the clock differences we reported.

Several limitations of our approach should be noted. First, we analyzed a total of nine longitudinal intervention datasets, which may not accurately capture the entire field of research. Second, sample sizes varied considerably across the datasets we analyzed (ranging from 14 to 92 subjects), which directly affects the statistical power of each statistical analysis. Intervention duration and methylation follow‐up times also varied a notable amount (ranging from 8 weeks to 2 years). Third, the datasets analyzed here were deposited under seven distinct preprocessing pipelines (Table 1), so effect‐size magnitudes should be compared across datasets with caution, though comparisons among clocks within a dataset are unaffected, as all derive from a single identically processed matrix. Finally, because no anti‐aging intervention to‐date has ever been definitively shown to extend human lifespan, we lack a true gold‐standard positive control for anti‐aging effects. Our “true positive” datasets are based on interventions with strong prior evidence for health benefits, but their effects on longevity remain to be established. More broadly, a robust change in epigenetic clock score does not necessarily translate to a change in lifespan or healthspan. The criteria proposed here are intended to distinguish biological signals from noise, but they do not address whether that signal is causally linked to longevity. Epigenetic clocks are predictive biomarkers, but their value as surrogate endpoints for lifespan will ultimately require validation against mortality outcomes in long‐term cohort studies.

In the future, this work should be extended to a larger, more systematic evaluation of intervention datasets as they become publicly available. The development of standardized clock batteries for clinical trials would help reduce publication bias and improve field‐wide trust in epigenetic clock results. Ultimately, the final validation for a biomarker's ability to detect a genuine change in biological age will require a ground‐truth health extending intervention, with longitudinal methylation sampling and mortality data. For epigenetic clocks to become standard tools in aging intervention research, the field will require a shared framework for distinguishing probable biological signal from statistical noise. The three criteria and effect‐size average proposed here offer one starting point, which we expect will be refined and expanded as more longitudinal intervention datasets become available.

## 4 Methods

### 4.1 Data Acquisition and Preprocessing

Where available, DNA methylation data were downloaded as beta values from public repositories, specifically the Gene Expression Omnibus (GEO) database (NIH) or the European Molecular Biology Laboratory's European Bioinformatics Institute (EMBL‐EBI). See Table 1 for per‐dataset processing pipelines. For datasets where methylation beta values were not directly available (E‐MTAB‐8956, E‐MTAB‐12527), we retrieved the raw fluorescence intensity files (idat files). These raw files were subsequently processed and converted into methylation beta values using the minfi package in R (version 1.48.0), following Normalization of Oligonucleotide Arrays by Background Subtraction (NOOB) and Quantile Normalization. DNA Methylation datasets used either Illumina 450 k or Illumina 850 k array platforms.

Phenotypic data was not directly modified; rather, six additional curated columns were appended to the phenotypic data frame. The six columns were adapted from the source data and included sample ID, subject ID, sex, age, group (control vs. subject), and time (in days). This step was done for all datasets to ensure standardized and replicable data handling in downstream analysis.

In cases of partially missing CpG probes, mean imputation was performed within the subject cohort. Missingness % was calculated for each dataset as the number of probes missing entirely from the dataset relative to the array type for the dataset. Entirely missing probes were left as NA because they are imputed identically at both time points, and they do not impact the longitudinal deltas.

### 4.2 Datasets

For datasets containing multiple timepoints, only two timepoints were selected for the analysis: the pre‐intervention baseline sample and a post‐intervention follow‐up sample. For these datasets, the time point selected for the follow‐up sample was always the first follow‐up time point. This approach was applied to maintain consistency and reduce complexity in longitudinal comparisons. Dataset GSE74548 was subset to include only female participants with the MTHFR 677CC genotype, aligning with the significant findings reported by (Sae‐Lee et al. 2018). Intensive surgery, for the case of dataset GSE142536, includes elective colorectal surgery, elective hip replacement surgery, and emergency hip surgery following fracture (Sadahiro et al. 2020).

### 4.3 Epigenetic Clock Calculation

We calculated scores for 12 well‐established epigenetic clocks, as summarized in Table 2. These clocks include both first‐generation clocks trained to predict chronological age (e.g., Hannum, Horvath MT, Horvath SB) and newer generation clocks trained to predict mortality risk or rate of aging (e.g., GrimAge, PhenoAge, DunedinPACE). We also calculated the PC version of these clocks, where available. Epigenetic clock scores for the two Horvath clocks, Hannum, PhenoAge, and DunedinPoAm38 were computed from the methylation beta values using the MethylCIPHER R package (version 0.20, https://github.com/HigginsChenLab/methylCIPHER). PC clock scores were calculated using the PC clocks package (https://github.com/HigginsChenLab/PC‐Clocks). GrimAgeV1 was calculated with a custom R function adapted from the biolearn python package (https://bio‐learn.github.io/). DunedinPACE was calculated using the DunedinPACE R package (version 0.99.0, https://github.com/danbelsky/DunedinPACE).

**TABLE 2:** Summary of the epigenetic clocks used in this study.

Clock	Trained to predict…	Training tissue	# of CpGs
Hannum (Hannum et al. 2013)	Chronological age	Whole blood	71
Horvath multi‐tissue (MT) (Horvath 2013)	Chronological age	51 tissues/cell types	353
Horvath skin and blood (SB) (Horvath et al. 2018)	Chronological age	Skin, blood, cultured cells	391
PhenoAge (Levine et al. 2018)	Mortality risk via 9 clinical chemistry marker proxies	Whole blood	513
GrimAge (Lu et al. 2019)	Mortality risk via 7 DNAm plasma‐protein surrogates + DNAm pack‐years + age + sex	Whole blood	1030
DunedinPoAm38 (Belsky et al. 2020)	Pace of aging	Whole blood	47
DunedinPACE (Belsky et al. 2022)	Pace of aging	Whole blood	173
PC Hannum (Higgins‐Chen et al. 2022)	Chronological age via Hannum	Whole blood	78,464
PC Horvath multi‐tissue (Higgins‐Chen et al. 2022)	Chronological age via Horvath MT	Whole blood	78,464
PC Horvath skin and blood (Higgins‐Chen et al. 2022)	Chronological age via Horvath SB	Whole blood	78,464
PC PhenoAge (Higgins‐Chen et al. 2022)	Mortality risk via PhenoAge	Whole blood	78,464
PC GrimAge (Higgins‐Chen et al. 2022)	Mortality risk via GrimAgeV1	Whole blood	78,464

### 4.4 Statistical Analysis

The primary outcome measure, age residual, was calculated for each subject by regressing predicted epigenetic age (DNAmAge) on chronological age by using the following linear model:

$$\text{resid}\left(\mathrm{lm}\left(\text{DNAmAge}\sim \mathrm{Age}\right)\right).$$

If subject‐level age was unavailable (only GSE73115), residuals were calculated using Zhang et al. 2019 instead of age. Models were built in R using the stats package (version 4.3.2). Paired *t*‐tests were conducted to compare age residuals before and after the intervention, paired by subject ID, also in R using the stats package. The change in epigenetic age residual (∆ EA resid) for one subject across the intervention was computed as follows:

∆EAresid=ageresidual after—ageresidual before.

These effect sizes were then scaled by normalizing the value by the clock population standard deviation, calculated from a large (4000+ samples) reference EPICv1 dataset.

∆EAresid scaled=∆EAresid/population standard deviation.

Correlations between clock scores were calculated using Spearman's correlation.

To calculate the average effect size (Figures 1, 2, and 5), we computed the average ∆ EA resid scaled across the 12 clocks on a per‐individual basis, averaged the individuals together for one final metric, and tested against zero with a one‐sample *t*‐test.

### 4.5 Cell Fraction Analysis

To assess the contribution of cell‐composition change to each intervention effect, cell fractions were estimated using the EpiDISH R package (version 2.18.0, https://bioconductor.org/packages/EpiDISH) (Teschendorff et al. 2017) with the 12‐celltype centroid reference *cent12CT.m* and the robust partial correlation (RPC) method (Luo et al. 2023). The 12 fractions correspond to naive and memory CD4+ T cells, naive and memory CD8+ T cells, regulatory T cells, naive and memory B cells, NK cells, monocytes, neutrophils, eosinophils, and basophils. A second residual was computed for each clock as resid(lm(DNAmAge ~ Age + 12 cell fractions)), paired Δ values were calculated subject‐by‐subject as before, per‐clock paired *t*‐tests were run on both residual definitions, and the effect size was scaled by the standard deviation of a population distribution with cell fractions residualised out. Attenuation was reported as (|raw effect| − |CF‐adjusted effect|)/|raw effect| × 100, with instances where the raw effect was below 0.05 HRS‐SD treated as missing.

### 4.6 Biological Age Simulation

For each individual i=1,…,N at timepoint t, the true latent biological age deviation is defined as

$$T_{\mathrm{it}}=\alpha_{i}+b_{i}\cdot t.$$

The baseline age deviation $\alpha_{i}$ is drawn from a normal distribution with mean 0 and standard deviation $\sigma_{\alpha }$. Individual aging slopes $b_{i}$ are drawn from a normal distribution with mean $\mu_{\beta }$ and standard deviation $\sigma_{\beta }$.

For clock *k* with reliability (ICC) $r_{k}$ the observed biomarker measurement is

$$X_{\mathrm{itk}}=T_{\mathrm{it}}+\varepsilon_{\mathrm{itk}}$$

where the measurement error variance $\varepsilon_{\mathrm{itk}}$ is drawn from a normal distribution with mean 0 and standard deviation $\sigma_{E,k}$. The variance of the clock scores $\sigma_{E,k}^{2}$ is derived from the definition of ICC scaled by the variance of the latent age at baseline ($\sigma_{T}^{2}$):

$$\sigma_{E,k}^{2}=\sigma_{T}^{2}\cdot \frac{1-r_{k}}{r_{k}}.$$

The intervention effect δ is applied as a uniform shift to all individuals at t=1 (post intervention). Simulation parameters are defined in Table 3.

**TABLE 3:** Latent age model parameters.

Parameter	Description	Value
N	Number of individuals	200
$\sigma_{a}$	Baseline age deviation SD	5 years
$\mu_{b}$	Mean slope	0
$\sigma_{b}$	Individual slope variability SD	3 years
δ	Intervention effect (panels B & C)	‐1 year

### 4.7 Multiple Testing Correction

We performed Bonferroni and Benjamini‐Hochberg corrections to account for multiple hypothesis testing (Table S2). Calculations were performed in R using a custom‐built function.

## Author Contributions

A.H.‐C., D.S.B., R.S. conceived the project and study design. D.S.B. developed the analysis pipeline and identified relevant datasets, and R.S., J.F.A., J.K., and J.G. assisted in dataset and code preparation. A.H.‐C. and D.S.B. wrote the manuscript. All authors helped edit and prepare the manuscript for submission.

## Funding

The work was supported by the National Institute on Aging under grant number R01AG060110, R01AG065403, and T32AG019134. It was also supported by the Impetus Grant from the Norn Group (R.S.), the Gruber Science Fellowship at Yale University (R.S.), and the Thomas P. Detre Fellowship Award in Translational Neuroscience Research from Yale University (to A.H.‐C.).

## Conflicts of Interest

R. Sehgal and A.H.‐C. are named as co‐inventors of a DNA‐methylation biomarker not utilized in the present study. A.H.‐C. has received consulting fees from TruDiagnostic and FOXO Biosciences. R. Sehgal has received consulting fees from TruDiagnostic, LongevityTech.fund and Cambrian BioPharma. The other authors do not declare any conflicts of interest.

## Supporting information


**Figure S1:** Three interventions datasets and the average clock changes across 12 epigenetic clock models for subject and control groups. Effect sizes are scaled to the standard deviations from a reference dataset.

**Figure S2:** Sensitivity of the per‐subject 12‐clock average to clock selection. For each dataset, the per‐subject average change in scaled epigenetic age residual is shown for three clock sets: all 12 clocks (black), the six reliable clocks (five PC clocks + DunedinPACE, blue), and the range across the twelve leave‐one‐out sets of 11 clocks (gray). (a) Effect size with 95% CIs (all−12, reliable) or the range of leave‐one‐out estimates. (b) Significance as –log_10_ *p*; shaded region marks *p* ≥ 0.05. Directional significance was unchanged in 137 of 144 leave‐one‐out cases.

**Figure S3:** Cross‐clock correlations from three longitudinal control datasets. (A) Correlation (*r*) between clock residuals at the baseline time point. (B) Correlation (*r*) between the change in clock residuals from baseline to follow up.

**Figure S4:** Pairwise correlations of changes in clock residuals between DunedinPACE and PC vs Original clocks. Pairwise Spearman correlations between DunedinPACE and five PC clocks (left) or heir corresponding original versions (right) for delta age esiduals (follow‐up minus baseline) across three longitudinal control datasets (GSE123914, GSE61151, GSE73115). Each point represents one clock pair in one dataset (5 pairs × 3 datasets = 15 points per violin). Points are colored by clock family. Dashed line indicates *r* = 0.

**Figure S5:** Cell composition and cell‐fraction‐adjusted intervention effects. (A) Mean EpiDISH‐estimated leukocyte composition (12 cell types) per dataset. The two non‐blood datasets (bold) are excluded from cell‐fraction adjustment, as EpiDISH is a whole‐blood reference: GSE98876 (purified CD3+ T‐cells; ~0% neutrophils, signal misassigned to monocytes/memory‐B) and GSE149747 (saliva; epithelial cells unrepresented, forced to a ~74% neutrophil profile). (B) Paired *t*‐tests on the change in age residual (Δ = follow‐up − baseline) for 12 clocks across the seven whole‐blood datasets. Effect sizes are standardized by a reference‐population SD (1.0 = one SD): filled black = unadjusted (clock ~ cAGE); open orange = additionally adjusted for the 12 EpiDISH cell fractions (CD4Tnv, CD4Tmem, CD8Tnv, CD8Tmem, Treg, Bnv, Bmem, NK, Mono, Neu, Eos, Baso). Lines show attenuation; italic labels give median attenuation across clocks. Dashed line marks zero.

**Figure S6:** Each of the 144 clock‐dataset pairs, plotted as per‐clock imputation versus |effect size|, both expressed relative to their dataset mean. Black solid line shows a linear fit to the data.

**Table S1:** The impact of leave‐one‐out (LOO) sensitivity analysis on the results of average intervention effect size. LOO *p*‐value range shows the minimum and maximum resulting *p*‐value after dropping one clock from the average calculation. LOO call stable reports if any of these drops lead to a loss in significance.

**Table S2:** The impact of multiple hypothesis testing on individual clock effect size significance. For each clock result, we re‐assessed the significance based on a threshold set after either Benjamini‐Hochberg or Bonferroni correction. Significant results are signified by an asterisk in the table's cell.

**Table S3:** The impact of cell fraction attenuation on the effect size after residualizing each clock on Age + 12 EpiDISH cell fractions. The last row shows the median effect size across all clocks in a dataset, and this is what the discussion quotes. The final column shows median attenuation for a single clock across all datasets.

**Table S4:** The percentage of missing probes for each clock in each dataset. The Missingness column shows % missingness of CpGs across the entire array.

## Data Availability

All methylation array data in this study comes from publicly available sources, including the Gene Expression Omnibus (GEO) and the EMBL's European Bioinformatics Institute (EMBL‐EBI). Code to calculate all clocks is accessible at https://github.com/HigginsChenLab/methylCIPHER. All cleaned datasets, analysis, and simulation workflows can be found at https://github.com/dsborrus/WhenToTrustEpigeneticClocks.
