# Supplementary material for: When to Trust Epigenetic Clocks: Avoiding False Positives in Aging Interventions
Source: Aging Cell. 2026 Oct 8;25(10):e70756. doi: 10.1111/acel.70756 (PMC13650178; doi:10.1111/acel.70756)
Supplement: Supplementary file 1 [file ACEL-25-e70756-s002.pdf]

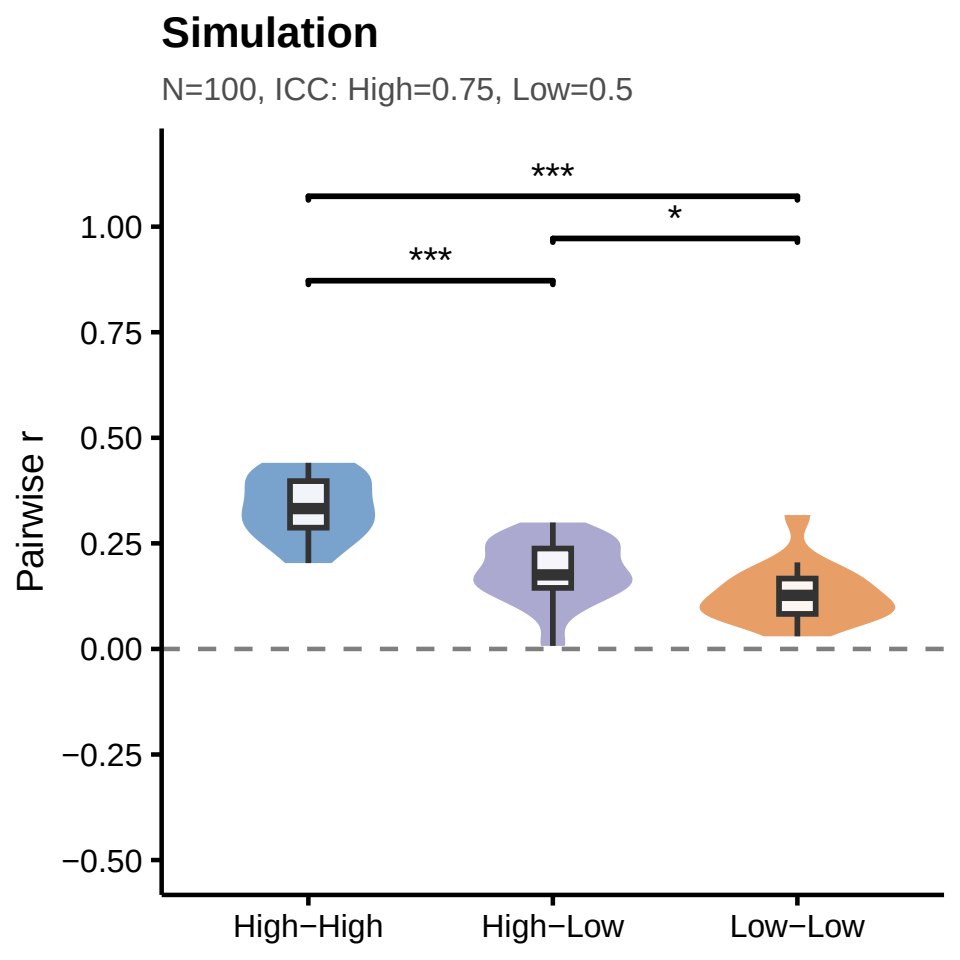

Simulation
N=100, ICC: High=0.75, Low=0.5
***
*
***
1.00
0.75
0.50
0.25
0.00
−0.25
−0.50
Pairwise r
High−High
High−Low
Low−Low
