# Supplementary material for: When to Trust Epigenetic Clocks: Avoiding False Positives in Aging Interventions
Source: Aging Cell. 2026 Oct 8;25(10):e70756. doi: 10.1111/acel.70756 (PMC13650178; doi:10.1111/acel.70756)

GSE149747 Diet, Exercise, Supplements 8 Weeks

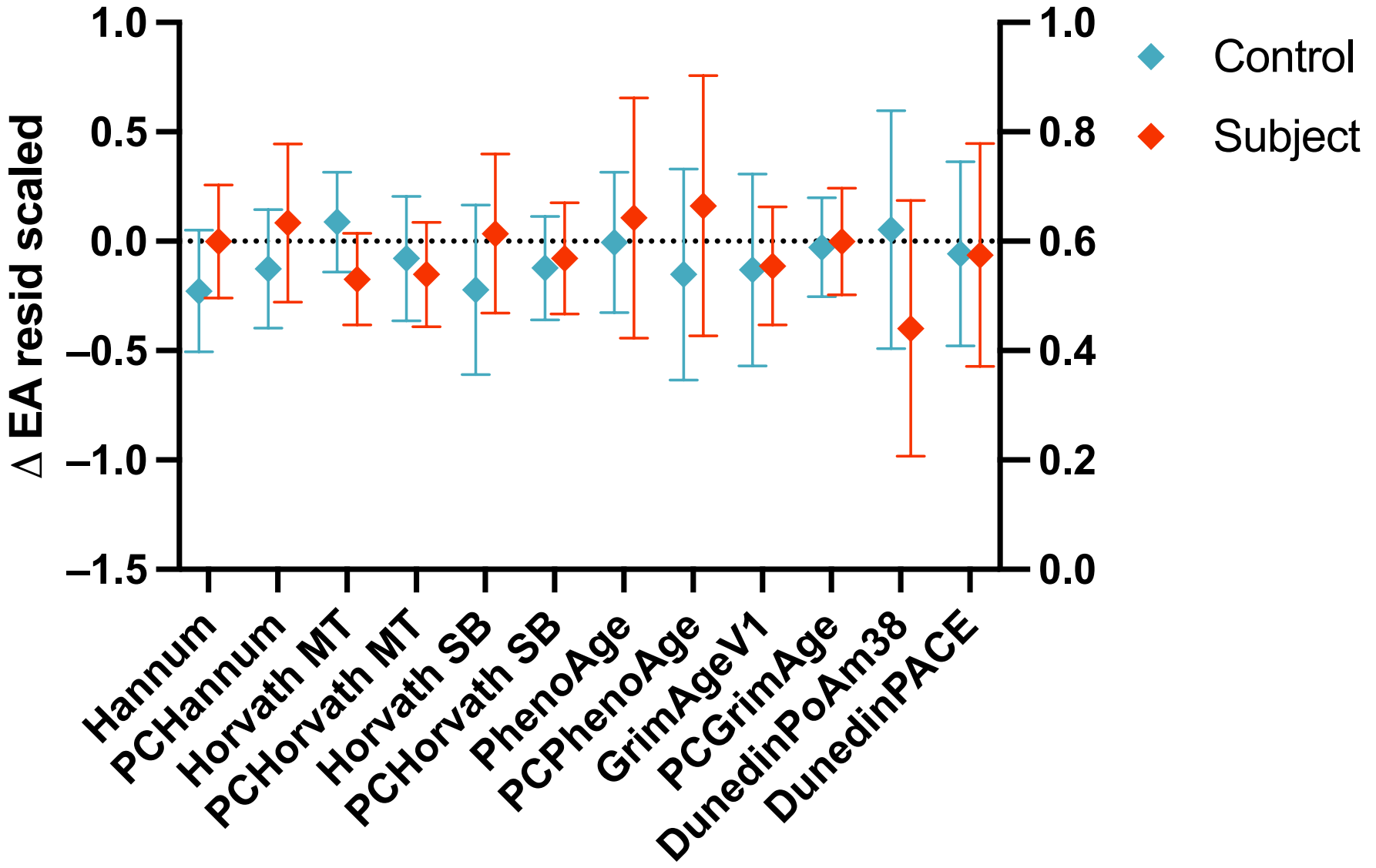

E-MTAB-8956 Low Carb Diet & Exercise 1.5 Years

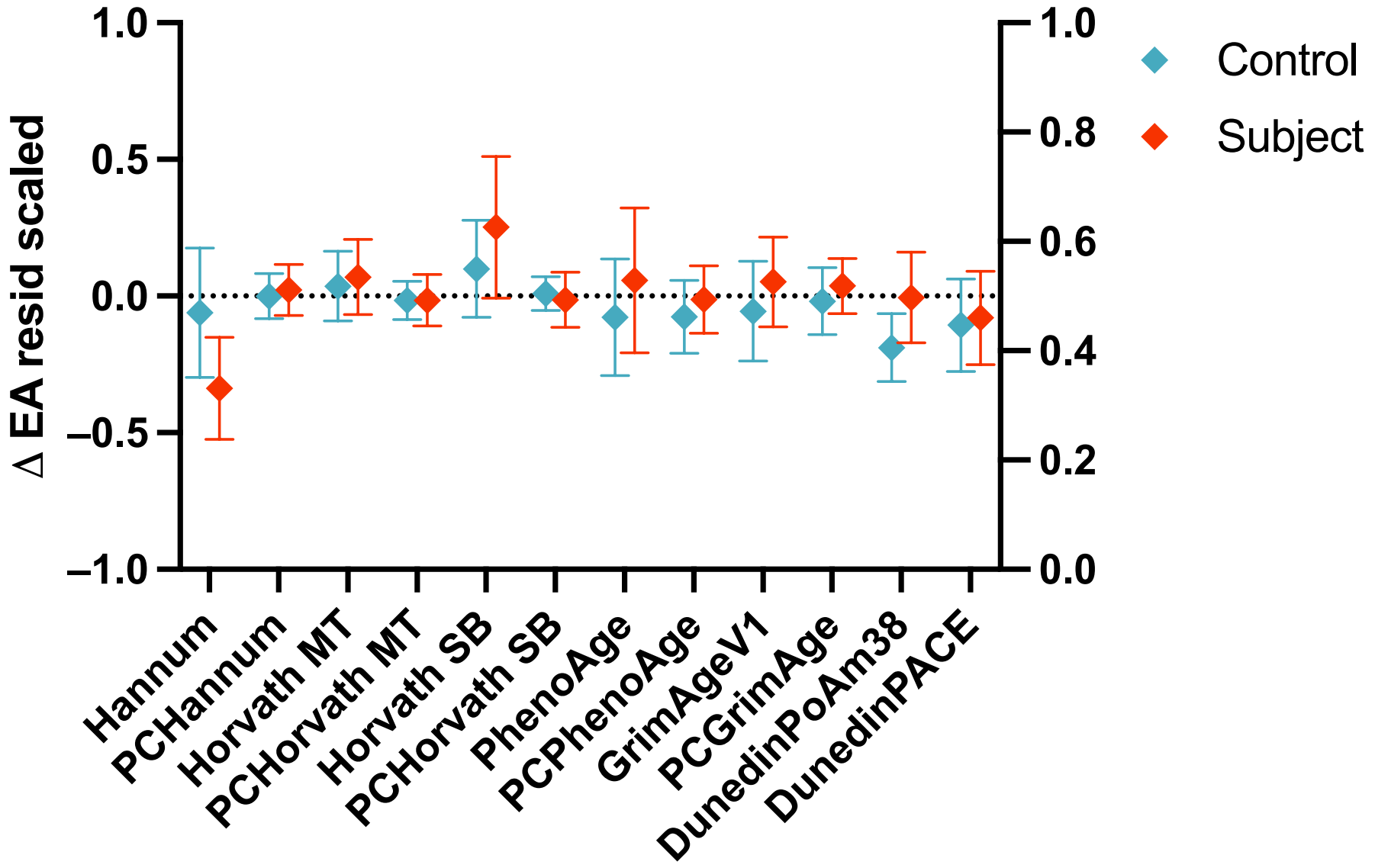

GSE74548 Folic Acid and Vitamin B12 2 Years

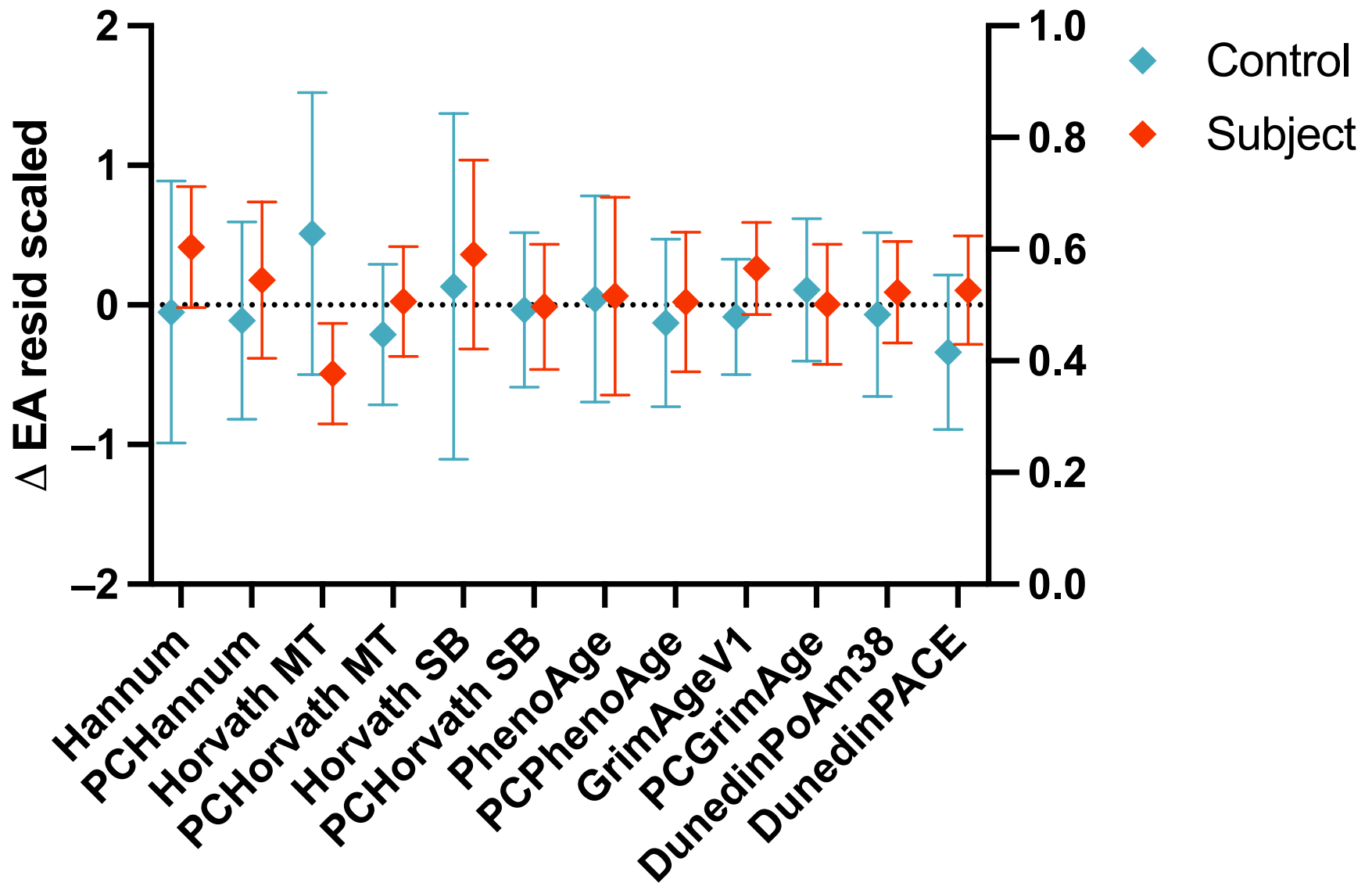

Supplement: Supplementary file 2 [file ACEL-25-e70756-s005.pdf]
