# Supplementary material for: When to Trust Epigenetic Clocks: Avoiding False Positives in Aging Interventions
Source: Aging Cell. 2026 Oct 8;25(10):e70756. doi: 10.1111/acel.70756 (PMC13650178; doi:10.1111/acel.70756)
Supplement: Supplementary file 3 [file ACEL-25-e70756-s007.pdf]

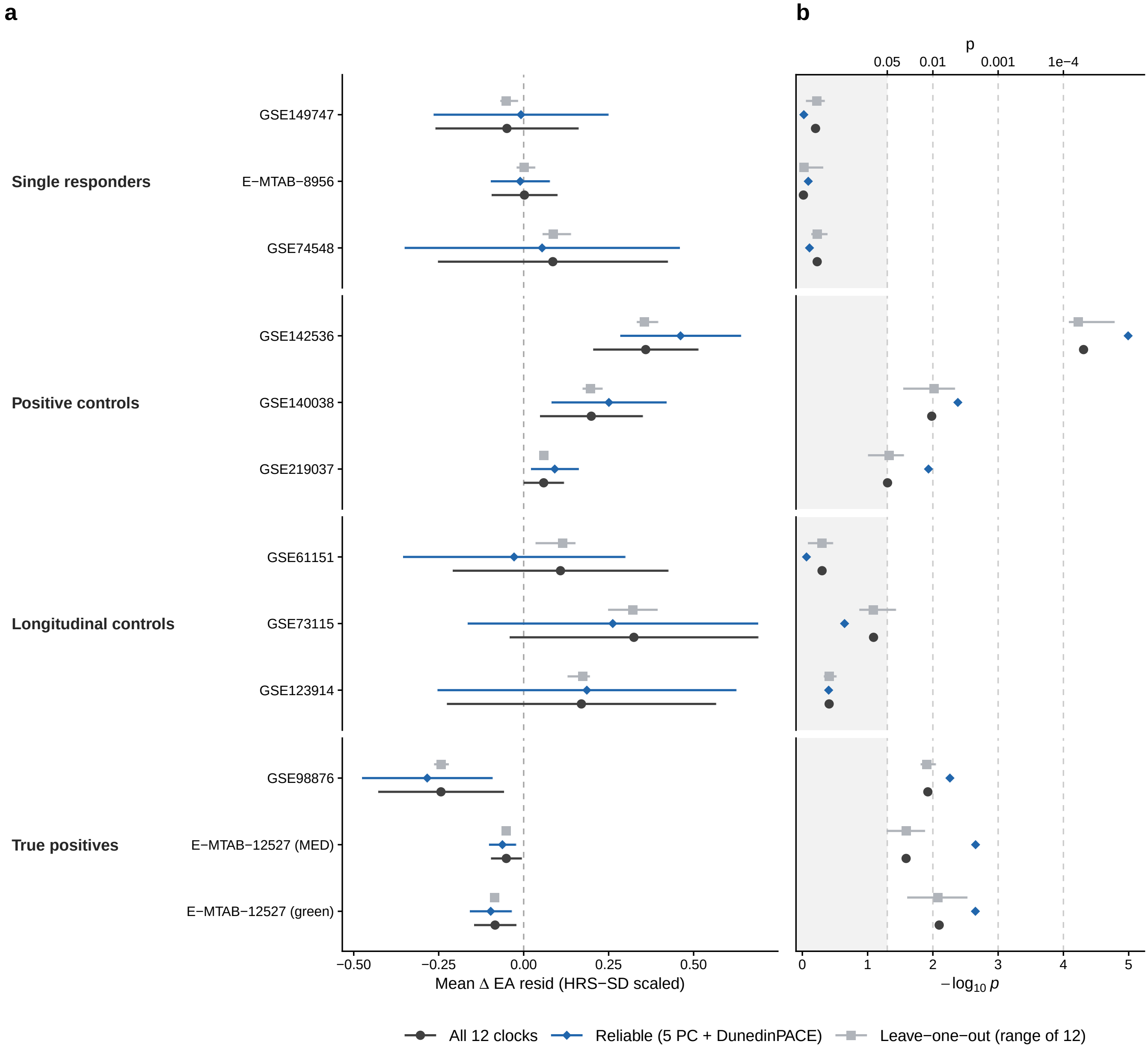

a
b
p
0.05
0.01
0.001
1e−4
Single responders
GSE149747
E−MTAB−8956
GSE74548
Positive controls
GSE142536
GSE140038
GSE219037
Longitudinal controls
GSE61151
GSE73115
GSE123914
True positives
GSE98876
E−MTAB−12527 (MED)
E−MTAB−12527 (green)
−0.50
−0.25
0.00
0.25
0.50
Mean Δ EA resid (HRS−SD scaled)
0
1
2
3
4
5
$-\log_{10} p$
All 12 clocks
Reliable (5 PC + DunedinPACE)
Leave−one−out (range of 12)
