# Supplementary material for: When to Trust Epigenetic Clocks: Avoiding False Positives in Aging Interventions
Source: Aging Cell. 2026 Oct 8;25(10):e70756. doi: 10.1111/acel.70756 (PMC13650178; doi:10.1111/acel.70756)
Supplement: Supplementary file 4 [file ACEL-25-e70756-s008.pdf]

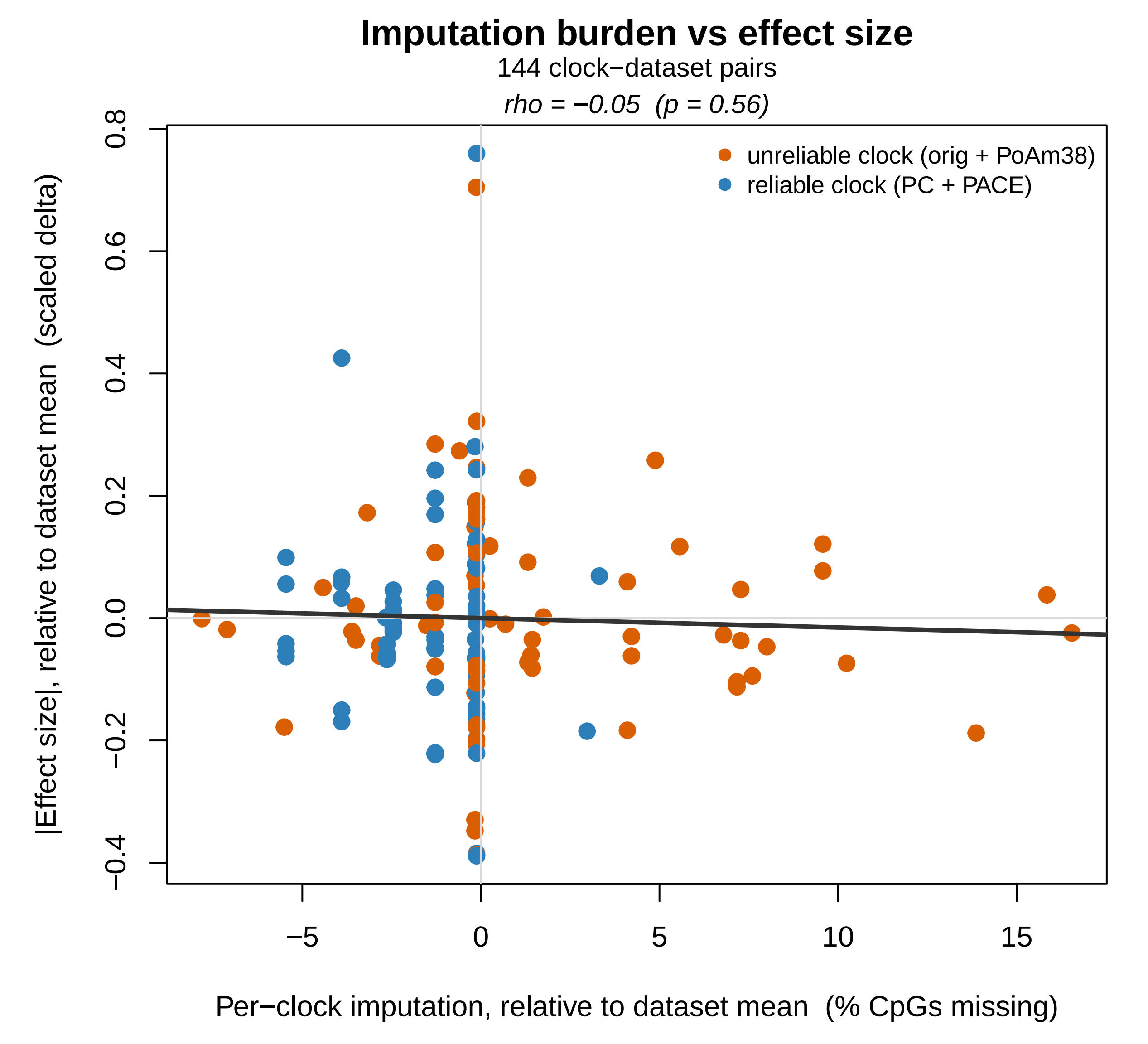
Imputation burden vs effect size
144 clock−dataset pairs
rho = −0.05 (p = 0.56)
unreliable clock (orig + PoAm38)
reliable clock (PC + PACE)
|Effect size|, relative to dataset mean (scaled delta)
0.8
0.6
0.4
0.2
0.0
−0.2
−0.4
−5
0
5
10
15
Per−clock imputation, relative to dataset mean (% CpGs missing)
