# Supplementary material for: When to Trust Epigenetic Clocks: Avoiding False Positives in Aging Interventions
Source: Aging Cell. 2026 Oct 8;25(10):e70756. doi: 10.1111/acel.70756 (PMC13650178; doi:10.1111/acel.70756)
Supplement: Supplementary file 5 [file ACEL-25-e70756-s009.pdf]

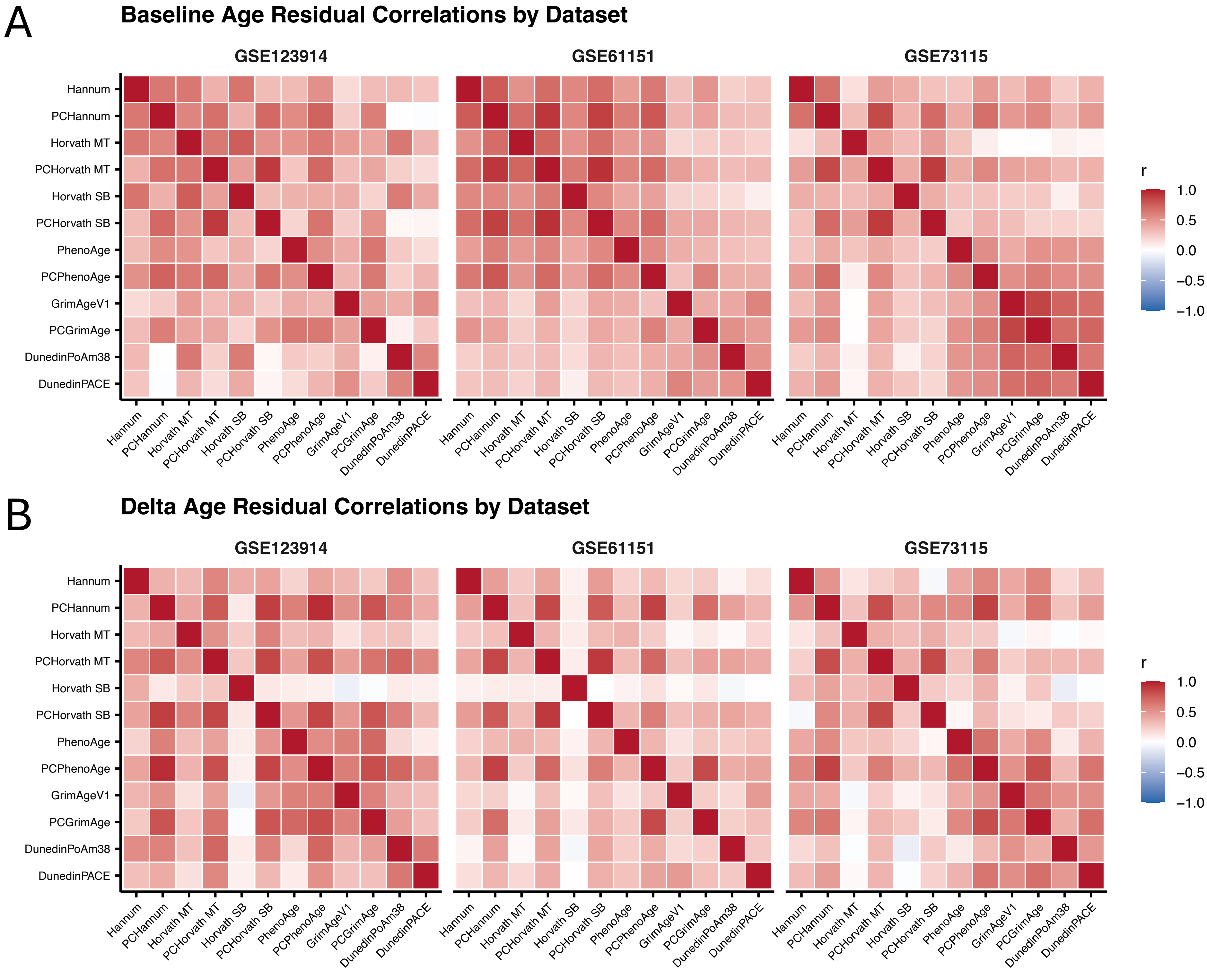
A
Baseline Age Residual Correlations by Dataset
B
Delta Age Residual Correlations by Dataset
GSE123914
GSE61151
GSE73115
Hannum
PCHannum
Horvath MT
PCHorvath MT
Horvath SB
PCHorvath SB
PhenoAge
PCPhenoAge
GrimAgeV1
PCGrimAge
DunedinPoAm38
DunedinPACE
r
1.0
0.5
0.0
−0.5
−1.0
