# Supplementary material for: When to Trust Epigenetic Clocks: Avoiding False Positives in Aging Interventions
Source: Aging Cell. 2026 Oct 8;25(10):e70756. doi: 10.1111/acel.70756 (PMC13650178; doi:10.1111/acel.70756)
Supplement: Supplementary file 6 [file ACEL-25-e70756-s010.pdf]

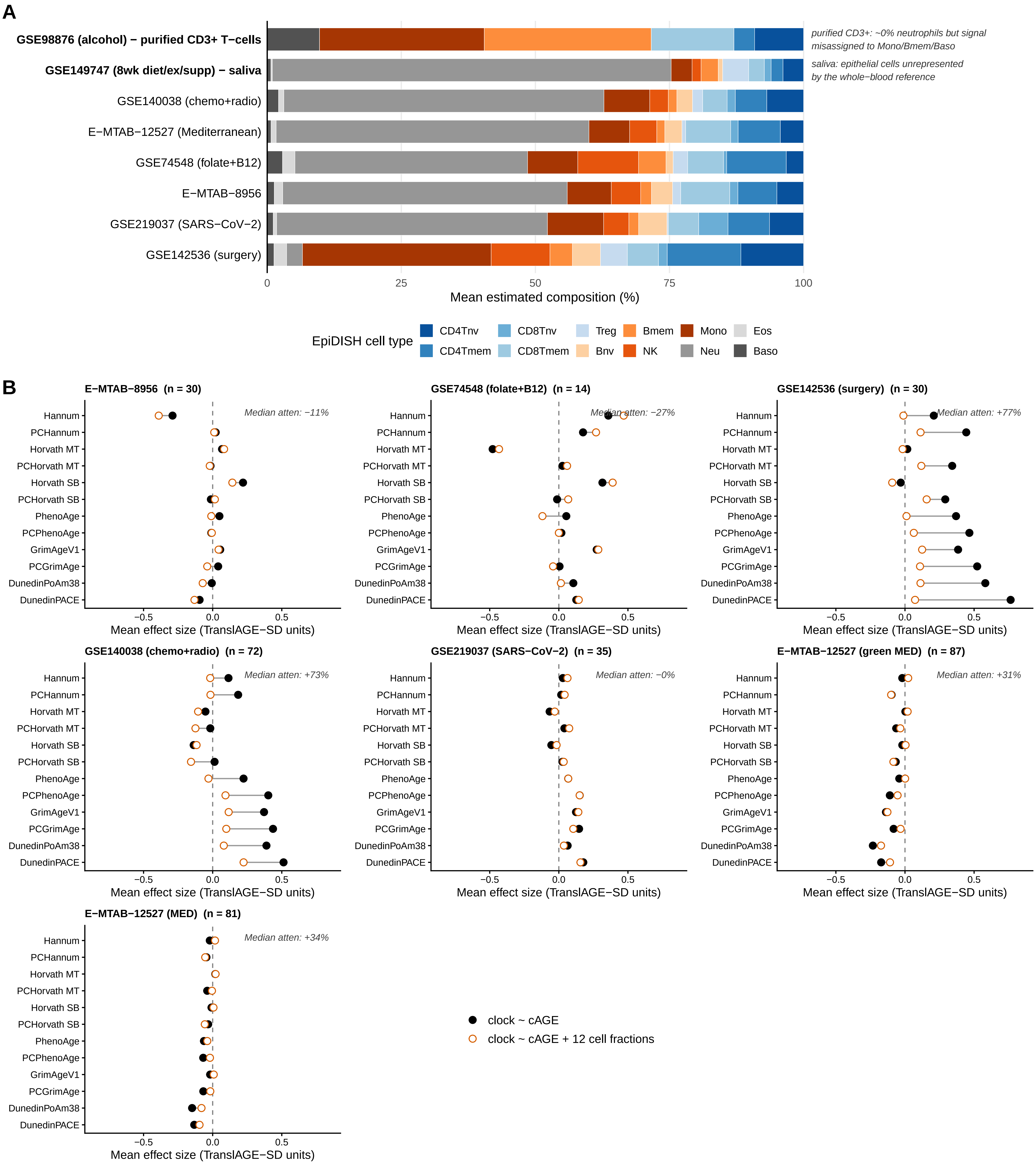
A
GSE98876 (alcohol) − purified CD3+ T−cells
GSE149747 (8wk diet/ex/supp) − saliva
GSE140038 (chemo+radio)
E−MTAB−12527 (Mediterranean)
GSE74548 (folate+B12)
E−MTAB−8956
GSE219037 (SARS−CoV−2)
GSE142536 (surgery)
purified CD3+: ~0% neutrophils but signal misassigned to Mono/Bmem/Baso
saliva: epithelial cells unrepresented by the whole−blood reference
0
25
50
75
100
Mean estimated composition (%)
EpiDISH cell type
CD4Tnv
CD4Tmem
CD8Tnv
CD8Tmem
Treg
Bnv
Bmem
NK
Mono
Neu
Eos
Baso
B
E−MTAB−8956 (n = 30)
Median atten: −11%
GSE74548 (folate+B12) (n = 14)
Median atten: −27%
GSE142536 (surgery) (n = 30)
Median atten: +77%
GSE140038 (chemo+radio) (n = 72)
Median atten: +73%
GSE219037 (SARS−CoV−2) (n = 35)
Median atten: −0%
E−MTAB−12527 (green MED) (n = 87)
Median atten: +31%
E−MTAB−12527 (MED) (n = 81)
Median atten: +34%
Hannum
PCHannum
Horvath MT
PCHorvath MT
Horvath SB
PCHorvath SB
PhenoAge
PCPhenoAge
GrimAgeV1
PCGrimAge
DunedinPoAm38
DunedinPACE
−0.5
0.0
0.5
Mean effect size (TranslAGE−SD units)
clock ~ cAGE
clock ~ cAGE + 12 cell fractions
